# A Novel Method for Measuring the Timing of Heart Sound Components through Digital Phonocardiography

**DOI:** 10.3390/s19081868

**Published:** 2019-04-19

**Authors:** Noemi Giordano, Marco Knaflitz

**Affiliations:** Dipartimento di Elettronica e Telecomunicazioni, Politecnico di Torino, 10129 Torino, Italy; noemi.giordano@polito.it

**Keywords:** phonocardiography, ECG, heart sounds

## Abstract

The auscultation of heart sounds has been for decades a fundamental diagnostic tool in clinical practice. Higher effectiveness can be achieved by recording the corresponding biomedical signal, namely the phonocardiographic signal, and processing it by means of traditional signal processing techniques. An unavoidable processing step is the heart sound segmentation, which is still a challenging task from a technical viewpoint—a limitation of state-of-the-art approaches is the unavailability of trustworthy techniques for the detection of heart sound components. The aim of this work is to design a reliable algorithm for the identification and the classification of heart sounds’ main components. The proposed methodology was tested on a sample population of 24 healthy subjects over 10-min-long simultaneous electrocardiographic and phonocardiographic recordings and it was found capable of correctly detecting and classifying an average of 99.2% of the heart sounds along with their components. Moreover, the delay of each component with respect to the corresponding R-wave peak and the delay among the components of the same heart sound were computed: the resulting experimental values are coherent with what is expected from the literature and what was obtained by other studies.

## 1. Introduction

Physiologically, the mechanical movement of the heart, and in particular the closing of the cardiac valves during each heartbeat, causes vibrations in the myocardium wall, which reflect as audible sounds, known as heart sounds [1,2]. In each cardiac cycle, two main heart sounds are expected to subsequently occur, namely the first (S_1_) and second (S_2_) heart sounds, corresponding to the closing of the mitral and tricuspid valves, and of the aortic and pulmonary valves, respectively. Beyond them, two low-frequency sounds, named third (S_3_) and fourth (S_4_) heart sounds, may be present, in addition to murmurs generated by sudden flow turbulence, often associated to pathological conditions [1,3].

Heart sounds auscultation by means of a stethoscope is a traditional routine screening tool in clinical practice, mainly due to its simplicity, speed, and low cost, combined with a high diagnostic value, in particular in case of valvular and vascular diseases. The intrinsic subjectivity and inaccuracy of the traditional methodology, as well as the diffusion of echocardiography, has led in recent years to a progressive decrease of its importance [2,3,4,5]. 

Higher effectiveness and objectivity may be obtained exploiting a microphone transducer to provide a noninvasive recording of cardiac acoustic vibrations, and thus an electrical trace of the heart’s mechanical behavior. The latter technique takes the name of phonocardiography. Transforming heart sounds into a biomedical signal, usually referred to as phonocardiogram (PCG), opens a wide range of possibilities, enabling the usage of signal processing and data analysis techniques to obtain a quantitative understanding of the heart sounds [2,6].

Being the PCG signal non-stationary and typically affected by a wide range of artifacts, its processing and interpretation is not a trivial issue. A critical step of all PCG signal-processing tools is the segmentation of heart sounds, i.e., the partitioning of the signal into segments corresponding to S_1_, systole, S_2_ and diastole intervals. This task requires identifying on the signal the time of occurrence and duration of the main heart sounds, as well as their classification [5,6,7]. 

**Figure 1:**
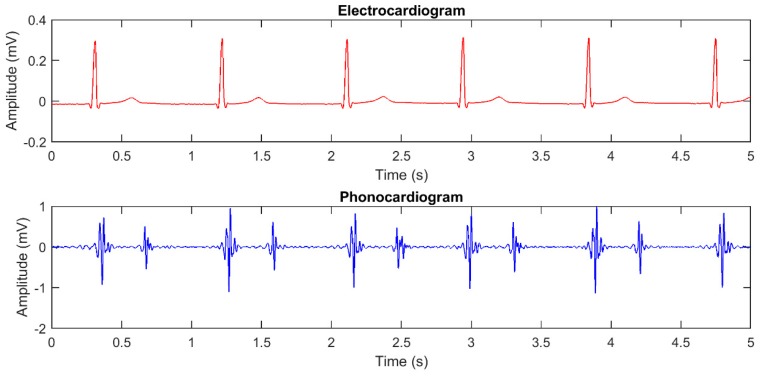
Plot of simultaneously recorded ECG and PCG signals, showing the temporal relationships among heart sounds and ECG waves.

Segmentation methodologies can be applied on the PCG signal only, or also involve the simultaneously recorded electrocardiogram (ECG) as a reference for the segmentation [6]. Figure 1 shows the plot of an ECG and of its corresponding PCG, presenting the two main heart sounds. 

A wide range of segmentation techniques has been described in the literature, including envelope-based approaches, wavelet-based approaches, machine learning techniques, methods based on time-frequency analysis and combinations of the mentioned approaches [5,6,7,8,9]. 

Nevertheless, no technique is as yet commonly in use in clinical practice. A robust and user independent identification of the beginning, peak and ending points of heart sounds is still a challenging task from the technical viewpoint [8,10]. The signal low amplitude, the presence of a wide range of artifacts and the intrinsic non-stationarity of the signal contribute to the difficulty. 

Moreover, the current literature usually focuses on the segmentation of the two main heart sounds, while much is still to be understood regarding more complex features of the PCG signal and their correlation with the physiology of the cardiac movements. 

An example is the analysis of the components of the main heart sounds. As aforementioned, the closing of two cardiac valves physiologically generates each heart sound. The closing is not exactly simultaneous, though, causing the presence of two components, separated by a delay, in the corresponding PCG [1]. The differentiation of the two components is infrequently discussed in the current literature, but it may widen the medical and technical knowledge related to heart sounds and open up new diagnostic possibilities.

The aim of this paper is to propose a user independent algorithm to identify the time occurrence of the two main components of the two main heart sounds within a single PCG recording. In particular, we describe a novel envelope-based segmentation algorithm aimed at identifying the instants of occurrence of each component of each heart sound with respect to the R-peak of the corresponding ECG signal. Finally, to test its reliability, the algorithm has been applied on a population of 24 healthy subjects. The timing of the components of S_1_ and S_2_ in the population are reported and compared with previously published data.

Before presenting the methodology we have designed for the measurement of heart sounds components timing, the following section provides a brief summary of the panorama of PCG segmentation techniques.

### State of the Art

Heart sound segmentation is a complex task and it is the object of a considerable amount of research activity. Heart sound segmentation and classification techniques are divided into direct and indirect, depending on whether an ECG is involved or not [6].

The most up-to-date methodologies for heart sound identification and classification were reviewed by Ismail et al. [5] in 2018. Those authors divided the available algorithms into time, frequency and mixed approaches. Segmentation methods in the time domain theoretically guarantee the most direct and reliable localization of heart sounds, but they need an intensive preprocessing of the signal, since they are highly sensitive to noise [5]. Therefore, most popular methodologies transform the PCG signal from the time domain into a different domain, where heart sounds are more easily recognized [6]. 

Envelope-based approaches are widely used, along with time-frequency techniques. The latter include wavelets, empirical mode decomposition, and time frequency representations [5,6,11]. All these possibilities proved to be suitable for obtaining average sensitivity values ranging from 73% to 99.4% [5,8,12,13,14,15,16,17,18,19,20]. To date, machine learning approaches are combined with traditional techniques to increase their automation and further improve their performance [5]. Also the combination of traditional techniques can increase the classification sensitivity, as proved by the 99.4% value obtained by Varghees et al. [8] with the application of a Hilbert transform to the Shannon entropy envelope of the signal.

The main advantage of envelope-based approaches resides in their capability of enhancing the identifiability of the heart sounds by converting them to a more suitable form for their localization in time, yet preserving a direct link with the time domain. The stated reason, along with the lack of the need for a training phase and thus for labeled data, makes envelope-based approaches the most widely used for PCG signal segmentation [5]. Specifically, Shannon energy, whose application to the detection of heart sounds components is the main focus of this work, is particularly appreciated as a technique to compute PCG envelopes, due to its unique feature of emphasizing medium intensity signals while attenuating the effect of low intensity components [21]. 

The application of Shannon energy as an envelope-based segmentation approach was first proposed as an envelope-based segmentation approach in 1997 by Liang et al. [21]. Their solution, achieving an average sensitivity of 94%, is still considered as a gold standard in the heart sound segmentation landscape [11]. The same authors later proposed an improvement of the algorithm by using discrete wavelet decomposition and reconstruction, thus obtaining a higher average sensitivity (97%) on a set of selected heartbeats [22]. 

Even though heart sound segmentation is a popular research subject, few is reported in the literature concerning the analysis of heart sound components. Moreover, most studies on the split between them use time frequency approaches, in some cases involving nonstationary decomposition, but they are mostly qualitative [23,24,25]. Sensitivity values of 96% are reported by Barma et al. [23] for the detection of the delay among second heart sound components.

The use of approaches based on the Shannon energy envelope for the localization of heart sounds components is even more unusual [25], because most heart sound segmentation techniques focus on the heart sound in the overall and thus use sliding windows with a 50% overlap, which conceal the presence of components.

## 2. Materials and Methods

The goal of this section is to provide an overview of the acquisition system used to record ECG and PCG signals as well as to describe the algorithms devolved to signal processing and their experimental validation. 

### 2.1. Acquisition System

The proposed methodology requires the simultaneous acquisition of an ECG and a PCG signal. Recording is realized by means of a commercial acquisition system for biomedical signals (ReMotus^®^, IT-MeD, Turin, Italy). This system may acquire up to four different channels. Signals are sampled simultaneously with a sampling frequency equal to 1 kHz and then converted by a 24-bit A/D converter (ENOB 17.25). The 3-dB bandwidth of the system spans from DC to 262 Hz and the input referred noise ranges between 2.9 µV_rms_ and 0.6 µV_rms_ depending on the selected gain (1–12). The system is fully isolated for patient safety (CF type) and is connected via a USB port to a host PC where signals are displayed, stored, and processed. 

ECG recording is carried out by means of an active probe. Its input impedance is higher than 1 GΩ, gain is equal to 0 dB and bandwidth spans from DC to over 10 kHz, hence the band of the signal is limited by that of the recording system (262 Hz).

PCG recording relies on a microphone probe custom realized for this study. The microphone probe is composed of a condenser microphone and the necessary electronic circuits. It is housed into a 3D-printed plastic container shaped as a truncated cone, as the traditional stethoscope. The frequency response of the microphone probe spans from 2 Hz to 250 Hz and hence it is suitable to record PCG signals for analyzing the two main heart sounds, whose frequency content spans from 20 Hz to 200 Hz [26].

### 2.2. Electrodes and Microphone Positioning

Three disposable adhesive Ag/AgCl electrodes (one reference electrode, two recording electrodes) are used for ECG recording. They are positioned over the subject chest as to recreate the I standard lead.

The microphone probe position over the patient chest is not standard, because the best auscultation places for heart sounds depend on the subject. Hence, the examiner needs to find experimentally a position that grants a PCG signal of sufficient quality. The search for the right position is carried out while visualizing the signal on the host computer throughout the ReMotus^®^ proprietary software interface. 

**Figure 2:**
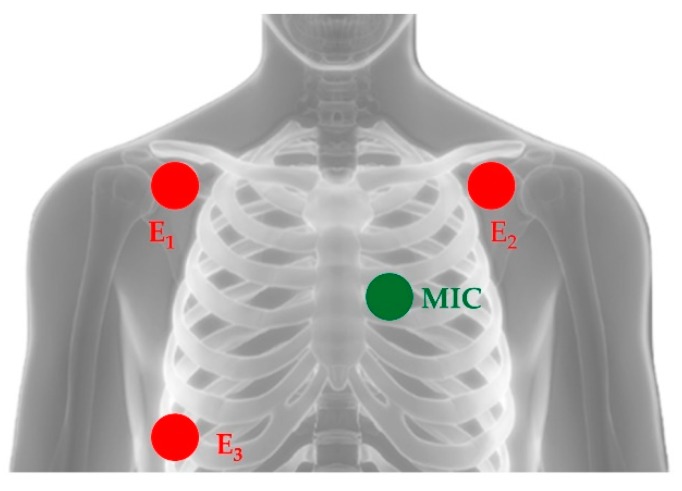
Typical positioning of electrodes for ECG recording (here denoted with abbreviations E_1_, E_2_ and E_3_) and of the microphone probe for the acquisition of the PCG signal (here denoted as MIC).

Figure 2 shows the typical positions of the three ECG electrodes and of the microphone probe. The latter is commonly located on the left hemithorax along one of the subject’s intercostal spaces. E_1_ and E_2_ are the recording electrodes while E_3_ is the reference electrode.

### 2.3. Signal Processing

**Figure 3:**
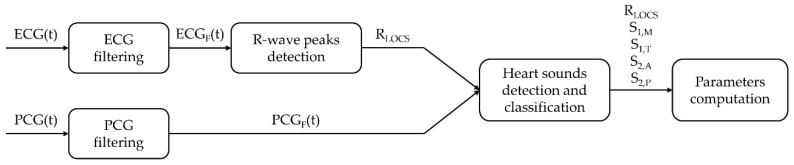
Block diagram of the overall processing.

The temporal locations of the R-peaks extracted from the ECG signal are used as a reference for the segmentation of the PCG signal as well as to identify the timing of the two main heart sounds and of their components. The processing is implemented in the Matlab^®^ environment. Figure 3 presents a block diagram of the overall algorithm. 

#### 2.3.1. ECG Filtering

ECG band-pass filtering is the first processing step, aimed at reducing the effect of the most common interferences as baseline wandering and high frequency noise. The filter was designed as the cascade of two Finite Impulse Response (FIR) filters of order 250, high-pass and low-pass, respectively with cut-off frequencies of 10 Hz and 35 Hz. This band was chosen because it contains the main frequency components of the QRS-complex [27]. 

The application of the cascade of two FIR filters of order 250 introduces a 250-sample delay in the ECG. Driven by the need for preserving the exact time relationships among the two recordings, the FIR filtering delay was compensated by eliminating the first 250 samples from the filtered ECG and the last 250 samples from the filtered PCG and signals were accordingly realigned. 

**Figure 4:**
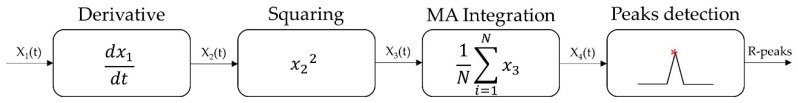
Block diagram of the implemented Pan-Tompkins algorithm.

A modified version of Pan-Tompkins algorithm [27] is then applied to the filtered ECG. This algorithm is a highly sensitive and energy efficient method based on linear and nonlinear filters for recognizing QRS-complexes, in particular R-waves, within an ECG recording. Figure 4 shows the main steps of the algorithm. 

A derivative filter is applied to the band-pass filtered ECG to enhance the signal high-frequency components. The derivative filter is then followed by a point-wise squaring and by the application of a moving average integrative filter with a window length of 150 ms. This length was chosen since it is close to the average duration of the QRS-complex in normal subjects. Peaks are detected on the resulting pulse-shaped signal through decision rules based on a double-step thresholding process aimed at increasing the algorithm sensitivity [27]. 

#### 2.3.2. PCG Filtering

The recorded PCG signal is first band-pass filtered. Since the frequency content of the main heart sounds is not yet universally agreed in the literature, the appropriate cut-off frequencies were experimentally defined by observing the Power Spectrum Density function of signals recorded on a population consisting of 24 healthy subjects. As a result, to obtain noise reduction yet preserving the main heart sounds components, we chose a band-pass Infinite Impulse Response (IIR) Chebyshev filter of order 5. Its band extends from 20 Hz to 100 Hz. The value of the low-pass cut-off frequency is particularly important because it is responsible for the reduction of heart murmurs, whose bandwidth typically spans from 200 Hz to 600 Hz [8], which otherwise may cause problems in segmentation. 

It should be highlighted that IIR filters do not provide a linear phase response, and hence may provoke distortions of the signal morphology in the time domain. As a solution to the stated drawback, zero-phase digital filtering was performed, i.e., the IIR filter was subsequently applied to the PCG recording both in the forward and in the backward directions. 

#### 2.3.3. PCG Segmentation

Among the wide range of segmentation methods described in literature, we preferred the implementation of an envelope-based technique because of its robustness towards noise in the same frequency band of heart sounds [10]. In particular, we compute the PCG envelope by means of its second order Shannon energy. This approach was already described in the literature for the segmentation of PCG signals, with promising results in terms of sensitivity and specificity [21,28].

Equation (1) presents the general formulation of second order Shannon energy (SE) [21,28]:

(1) $$E_{S}=-\frac{1}{N}\sum_{i=1}^{N}x^{2}\left(i\right)\cdot \log x^{2}\left(i\right)$$

where *x* is the normalized filtered PCG signal and *N* is the length (in samples) of the moving integration window, here set to 20 samples-20 ms with a sampling frequency of 1 kHz-to be compliant with the typical duration of the main components of heart sounds. 

Differently from what already presented in literature, where the integration window is generally moved with a 50% overlap of its own length, in this work the position of the integration window over the signal was incremented by one sample at a time, with the scope of preserving the original resolution of 1 ms, suitable for the discrimination of heart sounds components. 

It should be highlighted that higher order definitions of Shannon energy could provide a smoother envelope with a better noise reduction, but they could also conceal the presence of heart sounds components [28] whose detection is the goal of this work. 

With the scope of reducing the dependency of the detection on the signal quality, which may vary along the recording, a moving normalization is carried out as in Equation (2) [21,28]:

(2) $$E_{S}\left(t\right)=\frac{E_{S}\left(t\right)-\bar{E_{S}\left(t\right)}}{\sigma_{E_{S}\left(t\right)}},$$

where the mean $\bar{E_{S}\left(t\right)}$ and the standard deviation $\sigma_{E_{S}\left(t\right)}$ of the resulting Shannon Energy signal are computed over a 1-s-long sliding window. It should be highlighted that slightly longer normalization windows (e.g., 2-s-long) may be used as a security measure towards very low heart rates, with no statistically significant effect on the final result (α = 0.05). 

**Figure 5:**
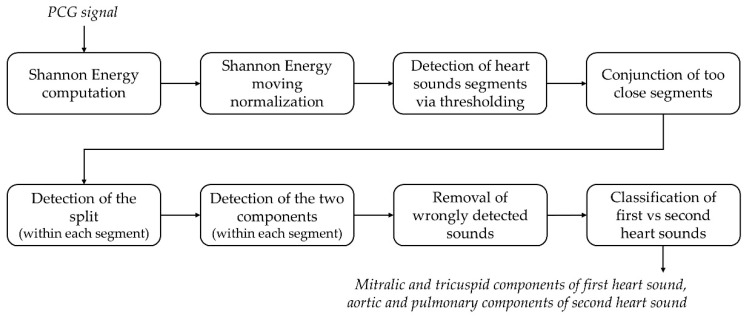
Block diagram of the heart sounds detection and classification algorithm.

Finally, all negative values are set to zero, thus obtaining a nonnegative envelope signal, characterized by the same length and resolution (1 ms) as the recorded PCG. The preservation of the initial time resolution is necessary to obtain a precise identification of the heart sounds main components. The detection of the two main heart sounds along with their components is performed on the Shannon energy signal based on amplitude and time thresholds. In particular, Shannon energy null values correspond to noise and should be discarded by means of an amplitude threshold [21,28]. Besides heart sounds, also murmurs and artifacts may cause positive peaks in the Shannon energy signal, but they can be deleted in a second phase thanks to the usage of time thresholds. Figure 5 shows the details of the detection algorithm.

The following steps describe the detection and classification of hearth sounds in detail, basing on an original approach involving amplitude and time thresholding:

(1) Heart sound segments are detected as the over-threshold SE points. The amplitude threshold is set to the 5% of the maximum SE value.
(2) Segments distant less than 10% of the mean RR-interval extrapolated from the ECG are joint together. The scope is to avoid the division of a single heart sound into two separated segments due to the presence of an under-threshold intercomponent split.
(3) The split is located within each segment as the deepest SE local minimum between the segment starting and ending point.
(4) First and second components are identified within each segment as the maximum SE value respectively between the segment starting point and the split, and between the split and the segment ending point.
(5) Removal of false positives. Basing on physiological temporal relationships among heart sounds, the following two decision rules are implemented to define false positives:
  (a) If two first components are outdistanced by less than the 20% of the mean RR-interval, the one with the lowest SE value is a false positive.
  (b) If two consecutive pairs of first components are both outdistanced by less than the 40% of the mean RR-interval, the first component with the lowest SE value is a false positive.
(6) Segments are classified as first or second heart sounds by means of temporal thresholding with respect to the corresponding R-wave peak, based on physiological ranges. In particular:
  (a) If the segment first component occurs between 50 ms before the R-peak and 18% of the corresponding RR-interval afterwards, then the segment is considered as a first heart sound and its components are respectively labeled as mitral and tricuspid.
  (b) If the segment first component occurs at least 18% of the corresponding RR-interval after the corresponding R-peak, then the segment is considered as a second heart sound and its components are respectively labeled as aortic and pulmonary.

**Figure 6:**
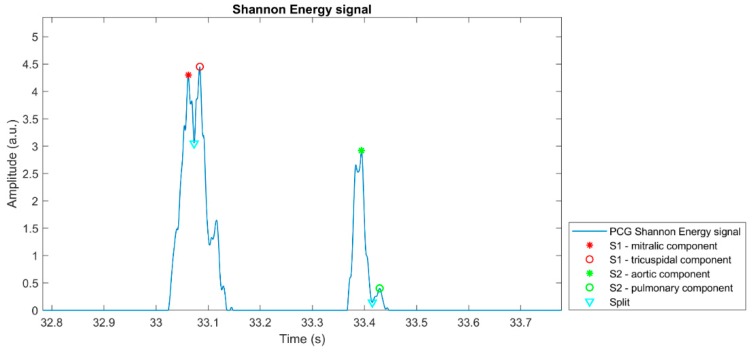
Example of the result of the application of the heart sounds detection algorithm on a heartbeat extracted from a 10-min PCG recording.

Figure 6 proposes a graphical representation of the outcome of the heart sounds segmentation algorithm, highlighting the positions of the detected heart sounds main components on the corresponding PCG Shannon Energy signal. The plot refers to a single heartbeat extracted from a 10-min recording.

#### 2.3.4. Extraction of Parameters

To validate the robustness of the implemented algorithm, we computed two parameters, namely the signal-to-noise ratio (SNR) of the filtered PCG, and the sensitivity of the algorithm in correctly identifying and classifying respectively the first and second heart sounds. 

Concerning the PCG SNR, it is defined as in Equation (3):

(3) $$SNR=20\log\frac{A_{S}}{4\;\sigma_{N}},$$

where $A_{S}$ is the peak-to-peak amplitude of the mean signal waveform and $4\;\sigma_{N}$ is a measure of the noise amplitude, i.e., the amplitude of the 95% band of the noise normal distribution, computed within the 70% and the 85% of the mean cardiac cycle since no heart sound is expected in this time window.

The sensitivity of the classifier is computed taking as a reference the number of the detected QRS-complexes, assuming that Pan-Tompkins is capable of correctly detecting all heartbeats within the recording. The latter assumption follows a careful manual verification by the authors on more than 3000 randomly selected heart cycles from ECG recordings on all the tested subjects, which resulted in a sensitivity as high as 100%, meaning that not a single heartbeat was lost. Therefore, sensitivity is defined as the ratio between the number of correctly detected heart sounds and the number of detected R-peaks. First and second heart sounds are considered separately. After identifying the temporal locations of the mitral and tricuspid components of the first heart sound and the aortic and pulmonary components of the second heart sound, we extracted some parameters describing the time relationships between the R-wave occurrence and different heart sound components. The following list shows the parameters we extracted:

- R-S_1,M_: the delay between the mitral component of the first heart sound and the corresponding R-peak;
- R-S_1,T_: the delay between the tricuspid component of the first heart sound and the corresponding R-peak;
- S_1,M_-S_1,T_: the delay between the tricuspid and the mitral components of the first heart sound (also referred to as “S_1_ split” in literature [2]);
- R-S_2,A_: the delay between the aortic component of the second heart sound and the corresponding R-peak;
- R-S_2,P_: the delay between the pulmonary component of the second heart sound and the corresponding R-peak;
- S_2,A_-S_2,P_: the delay between the aortic and pulmonary components of the second heart sound (also referred to as “S_2_ split” in literature [2]).

**Figure 7:**
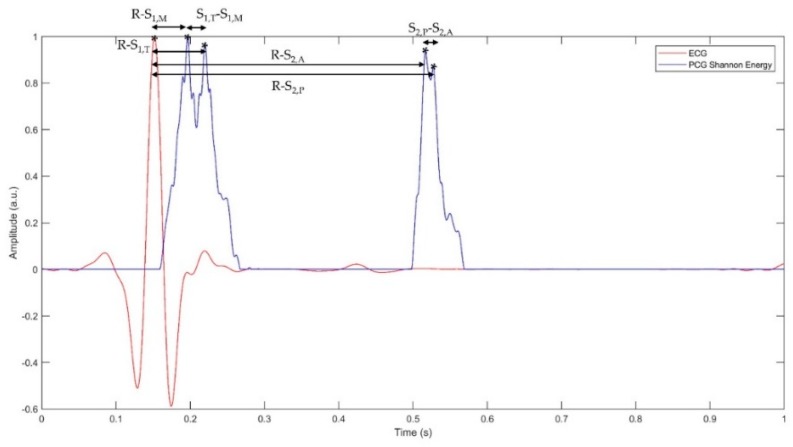
Representation of a filtered ECG signal and of the Shannon energy of the corresponding PCG signal within a single cardiac cycle.

Figure 7 shows representation of the parameters mentioned above within a cardiac cycle.

### 2.4. Sample Population

The algorithm was tested on a sample population composed of 24 healthy volunteers, different in terms of age, gender and body structure. Methods and extent of the study were thoroughly explained to participants, who gave their assent and signed an informed consent form. Since the study was observational, it could neither modify subjects’ health status nor expose subjects to any danger. Hence, we did not submit the protocol of the study to an ethical committee. Table 1 lists the Age, Gender, Height, Weight and Body Mass Index (BMI) of each specific subject of the population. 

The population included 11 female and 13 male subjects, with ages spanning from 18 to 83 years with an average of 38 years. Concerning body structure, the Body Mass Index (BMI) of subjects ranges from 17.7 kg m^−2^ to 30.5 kg m^−2^, height from 1.54 m to 1.87 m and weight from 50 kg to 85 kg, thus representing subjects ranging from underweight to overweight.

It should be highlighted that subject 05 had recurrent premature ventricular contractions (PVC), despite being healthy from a cardiological and general viewpoint. Since this condition is present in 1–4% of the overall world population [29], we valued the possibility of validating the robustness of the implemented algorithm towards PVCs as a plus. 

**Figure 8:**
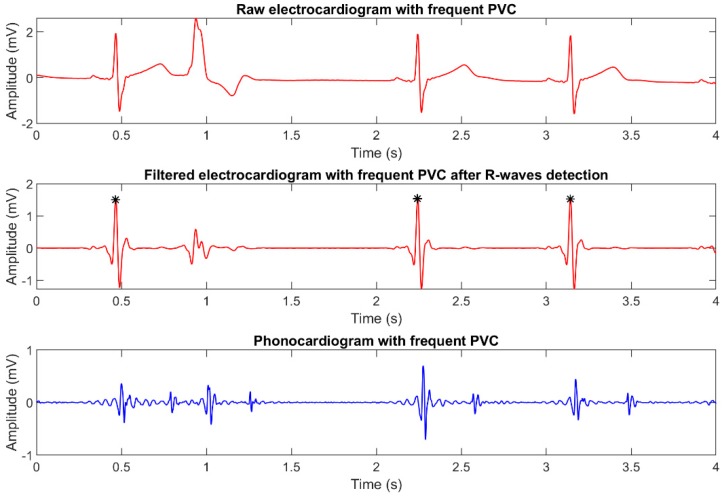
Plot of simultaneously recorded ECG and PCG signals, showing the presence of recurrent premature ventricular contractions.

Figure 8 presents an example of how PVCs affect the corresponding ECG and PCG recordings. As it can be seen in the plots, PVCs are strongly attenuated by digital filtering and are not detected by Pan-Tompkins algorithm, thus resulting in the discarding of the corresponding heart sounds, as expected. 

## 3. Results and Discussion

This section presents the results obtained by applying the algorithm object of this study to the population of healthy subjects described in the previous section. The aim is validating the methodology and comparing the range of variability of the parameters of interest with the literature. All values presented in this section refer to 10-min recordings. Three sample recordings (Signal S1, Signal S2 and Signal S3), characterized by decreasing SNR, are provided as supplementary material.

### 3.1. Algorithm Validation

The reliability of the proposed algorithm was evaluated considering two parameters: the PCG SNR and the algorithm detection sensitivity. Table 2 reports the values obtained on our sample population.

A high value of the SNR associated to the filtered PCG signal is a fundamental premise for the good functioning of the algorithm itself. Table 2 shows that, after filtering, all recorded PCG signals are characterized by a SNR higher than 10 dB, which we proved sufficient for a reliable postprocessing.

Table 2 also shows that S_1_ detection sensitivity spans from 97.3% to 100% (mean 99.6%, stdev 0.7%) and S_2_ detection sensitivity is always higher than 84.9% (mean 98.9%, stdev 3.2%). The overall average heart sounds classification sensitivity of the proposed algorithm, computed as the mean of the sensitivity values obtained for all the subjects considering both heart sounds, was found as high as 99.2%.

These results are better than most of those considered as the state-of-the-art. In fact, the gold standard algorithm presented by Liang et al. [21] proved an average sensitivity of 94% [21], whereas their later improvement [22] correctly recognized the 97% of the heart sounds. More recent studies [12,13,14,15,16,17,18,19,20], based on a wide range of different techniques, achieve sensitivity values up to 99%, with two exceptions [8,17] reaching 99.3% and 99.4%.

In terms of heart sound components, few detection algorithms [23,24,25,30] are reported in literature, especially concerning the first heart sound. When considering the approach described in this work, it must be observed that when a heart sound is correctly segmented both its components are identified. Hence, the sensitivity of detection of heart sounds components is equal to that reported for the corresponding heart sound. To our best knowledge, no sensitivity data of the detection of first heart sound components are currently available in literature for comparison. Regarding second heart sound components, the sensitivity we obtained (98.9%) is slightly higher than what previously reported (96% [23]). In all the subjects tested, the implemented algorithm achieves specificity values of 100%: no murmur nor noise component is misclassified as a heart sound.

### 3.2. First Heart Sound

Table 3 presents the numerical values of the parameters relative to the first heart sound: the delay of the mitral (R-S_1,M_) and the tricuspid (R-S_1,T_) components with respect to the corresponding R-peak and the delay among them (S_1,M_-S_1,T_).

It should be highlighted that mean and standard deviation values referring to a single subject are the result of the computation relative to a 10-minute recording, i.e., more than 500 heartbeats, depending on the heart rate and on the detection sensitivity. For a specific subject, the 95% confidence interval of the three latency values reported in the table has a width of at most 2 ms. This means that, when assessing the same subject in different days, changes of the mean value of a latency as small as 2 ms may be considered as statistically significant. 

Even though the population of interest is not broad enough to infer the physiological range of variability of the parameters reported, the values obtained are coherent to what reported in literature [1,25,30]. Indeed, the first heart sound is expected to occur few milliseconds after the R-wave peak and to last 140 ms [1], and the latency between its main components is expected to equal 20–30 ms [2]. 

### 3.3. Second Heart Sound

Similarly to what presented above concerning the first heart sound, Table 4 shows the experimental results related to the second heart sound. In particular, tabulated parameters are the delay of the aortic (R-S_2,A_) and pulmonary (R-S_2,P_) components with respect to the corresponding R-wave peak, and the delay among them (S_2,A_-S_2,P_). 

Also in the case of the components of the second heart sound, the experimental values we obtained are compliant with the physiological ranges reported in the literature and to the results obtained in former studies [1,23,24,25]. In particular, the second heart sound is expected to occur approximately 300 ms after the first one [1], which is coherent with the values reported in Table 4.

In terms of repeatability on a specific subject, the 95% confidence interval of the three latency values reported in the table has a width of at most 6 ms. This means that, when assessing the same subject in different days, changes of the mean value of a latency as small as 6 ms may be considered as statistically significant.

### 3.4. Clinical Implications

At this time, the instrumental non-invasive study of the heart relies principally on ECG, echocardiography, cardiac magnetic resonance and coronary CT. Since the delay between the closing of the atrioventricular valves or of the aortic and pulmonary valves is equal to a few tens of milliseconds [2,31], it follows that the time resolution that is needed for properly assessing the delay between heart sound components must be at least an order of magnitude less, i.e., 1–2 ms. None of the above mentioned techniques allows for the detection of heart valves closing with a time resolution as high as 1 ms or less.

In this study, we demonstrated that a very simple and inexpensive system is sufficient for assessing the hearth sound components. Since the procedure used to locate components is completely user independent, even a relatively inexperienced operator, as a caregiver, could place the ECG electrodes and the microphone on the patient chest to carry out the exam even at his domicile.

Several heart conditions could benefit from the assessment of heart sound components. In fact, myocardial inotropy affects the timing of both S_1_ and S_2_: a decrease in the development of ventricular pressure should cause an increment of R-S_1,M_ as well as of R-S_1,T_, while if only the right ventricle contraction force is diminished only R-S_1,T_ should increase. A similar effect should also be observed on R-S_2,A_ and on R-S_2,P_, but in this case the timing of the two components is also affected by the blood pressure in the aorta and pulmonary arteries. Hence, a quantitative characterization of the delay may prove beneficial for the diagnosis of bundle branch blockade and valvular diseases. In addition, some respiratory pathologies affect the S_2_ split due to the effect of inspiration. The timing of occurrence of each component within the cardiac cycle is also associated to the heart electro-mechanical coupling, whose quantitative measurement is a critical technical issue. 

A ready application of the analysis of heart sound components is the follow-up of patients suffering from congestive heart failure (CHF) at their domicile. CHF is a complex syndrome that results from any functional or structural impairment of the ability of ventricles to fill with or eject blood. This condition results in an inefficient pumping activity that compromises the oxygen delivery to metabolizing tissues [32,33].

Currently, the severity of CHF is mainly evaluated by ECG, echocardiogram, and by the evaluation of the plasma concentration of Brain Natriuretic Peptide [32]. Since CHF due to systolic dysfunction is associated with a reduced ejection fraction due to reduced inotropy, R-S_1,M_ and of R-S_1,T_ should be affected, as was proven by Oliveira Neto et al. [34] and by Efstratiadis et al. [35]. We hypothesize that, in patients suffering from CHF due to systolic dysfunction but well compensated, the evaluation of the two R-S_1_ subcomponents could allow for a simple and inexpensive monitoring of the patient status even at his domicile. 

The results presented in this work refer to normal subjects and aim at validating the reliability of the developed algorithm. The applicability of this methodology to pathological subjects and its real outcomes are still to be demonstrated.

## 4. Conclusions

The aim of this work is to propose an algorithm for the identification and classification of the two main components of each heart sound, namely the mitral and tricuspid components of first heart sound and the aortic and pulmonary components of second heart sound.

The algorithm correctly identified 99.2% of heart sounds and of their components in a test population of 24 healthy subjects. A comparison with the state of the art shows that the values of sensitivity obtained are higher than those achieved by most of the currently available techniques. Moreover, we performed a trustworthy quantitative characterization of the timing of occurrence of heart sounds components. To our best knowledge, results reported for the S_1_ components have not yet presented in the literature. 

The relative simplicity of this technique is as a plus in the heart sound segmentation panorama. Indeed, the developed algorithm does not base on highly computationally expensive techniques, such as Empirical Mode Decomposition and wavelets, nor it needs a training phase as it is typical of machine learning approaches. Even when compared to most other envelope-based algorithms, the proposed solution proves highly efficient because of its lack for a search-back phase in the heart sounds detection process.

The capability of quantitatively measuring the timing of heart sounds, of their components, and the latency among them may open to a wide range of clinical applications as the analysis of the heart electro-mechanical coupling, the relationship between right and left heart coordination, and the diagnosis of various cardiological, valvular, and respiratory diseases.

A limitation of this work is the validation of the methodology on a sample population of healthy subjects only. In fact, pathological conditions may cause alterations of the PCG waveforms that could compromise the reliability of the assessment of the timing of heart sound components. In the future, we will test the algorithm on pathological subjects to evaluate its reliability also in this condition.

## Figures and Tables

**Table 1:** Detail of the personal data describing the population of 24 healthy subjects on whom the methodology was tested.

Subject ID	Age	Gender	Height (m)	Weight (kg)	BMI (kg m^−2^)
subj_01	18	M	1.86	70	20.2
subj_02	56	F	1.70	80	27.7
subj_03	50	F	1.70	78	27.0
subj_04	76	F	1.62	80	30.5
subj_05	24	F	1.71	67	22.9
subj_06	23	F	1.63	60.5	22.8
subj_07	27	M	1.73	71	23.7
subj_08	24	M	1.74	65	21.5
subj_09	23	F	1.62	51	19.4
subj_10	25	M	1.75	70	22.9
subj_11	30	M	1.78	78	24.6
subj_12	19	M	1.80	68.5	21.1
subj_13	56	F	1.54	50	21.1
subj_14	53	M	1.78	85	26.8
subj_15	24	M	1.84	75	22.2
subj_16	26	M	1.85	78	22.8
subj_17	26	M	1.87	76	21.7
subj_18	27	M	1.80	65	20.1
subj_19	24	F	1.67	62	22.2
subj_20	55	F	1.60	52	20.3
subj_21	83	F	1.57	59	23.9
subj_22	83	M	1.65	74	27.2
subj_23	23	F	1.68	50	17.7
subj_24	24	M	1.80	78	24.1

**Table 2:** Algorithm validation: experimental results in terms of SNR of the filtered PCG and detection sensitivity.

Subject ID	Heart Rate (bpm)	PCG SNR (dB)	S_1_ Detection Sensitivity (%)	S_2_ Detection Sensitivity (%)
subj_01	66	33.7	100	100
subj_02	82	13.9	99.8	94.8
subj_03	80	16.1	99.7	97.6
subj_04	66	20.7	99.7	100
subj_05	68	28.3	100	99.8
subj_06	63	18.7	99.8	100
subj_07	63	27.2	100	100
subj_08	55	28.8	99.6	100
subj_09	78	15.0	100	100
subj_10	56	18.3	99.8	84.9
subj_11	56	30.2	100	100
subj_12	63	24.4	99.7	100
subj_13	84	14.7	99.8	100
subj_14	56	27.7	99.8	99.8
subj_15	65	16.2	100	99.8
subj_16	61	14.2	99.8	99.3
subj_17	67	20.7	97.3	99.1
subj_18	87	21.9	99.6	100
subj_19	86	19.0	97.6	99.1
subj_20	62	25.4	99.7	99.3
subj_21	65	12.5	99.5	99.7
subj_22	56	24.2	99.5	99.8
subj_23	76	14.4	99.2	99.9
subj_24	66	27.5	99.7	99.7
**Mean**	67.8	21.4	99.6	98.9
**Median**	65.6	20.7	99.8	99.8
**Std**	10.1	6.2	0.7	3.2
**IQR ^1^**	16.5	12.1	0.4	0.7
**Range 95%**	55.8–86.4	13.3–31.7	97.5–100	90.6–100

^1^ Interquartile Range.

**Table 3:** Numerical values of the parameters related to the first heart sound.

Subject ID	R-S_1,M_		R-S_1,T_		S_1,M_-S_1,T_	
	Value (ms)	Std (ms)	Value (ms)	Std (ms)	Value (ms)	Std (ms)
subj_01	45.0	3.2	64.3	2.8	19.3	5.3
subj_02	20.7	5.8	72.4	12.9	51.7	11.9
subj_03	33.3	8.9	63.9	15.9	30.6	17.4
subj_04	66.7	19.6	123.3	24.6	56.5	17.1
subj_05	36.7	8.4	50.2	10.6	13.6	6.0
subj_06	41.5	5.4	74.2	10.6	32.7	9.9
subj_07	49.9	6.1	69.5	15.8	19.6	10.2
subj_08	45.8	5.9	62.1	14.8	16.3	12.2
subj_09	17.9	6.8	72.1	8.1	54.1	9.7
subj_10	41.5	6.3	65.8	16.1	24.3	16.3
subj_11	39.2	4.8	60.3	18.7	21.1	16.4
subj_12	36.9	14.7	77.5	11.5	40.6	12.5
subj_13	39.2	14.7	60.5	7.2	21.3	12.9
subj_14	53.1	5.1	76.6	15.3	23.5	12.9
subj_15	32.4	5.4	57.1	23.8	24.7	22.5
subj_16	58.8	15.2	110.5	25.5	51.7	23.4
subj_17	23.8	14.3	87.4	20.5	63.6	19.7
subj_18	58.6	15.1	94.0	14.8	35.4	12.8
subj_19	43.6	11.6	68.7	28.0	25.1	26.5
subj_20	51.0	18.8	85.0	19.5	34.0	8.6
subj_21	61.2	14.1	98.5	13.4	37.3	18.4
subj_22	59.5	12.8	90.3	12.3	30.8	12.8
subj_23	42.1	13.2	68.6	14.0	26.5	14.6
subj_24	54.0	11.1	71.4	6.6	17.4	8.6
**Mean**	43.9		76.0		32.2	
**Median**	42.8		71.7		28.6	
**Std**	11.4		16.3		15.1	
**IQR** ^1^	17.1		22.8		18.6	
**Range 95%**	19.5–63.6		54.2–115.9		15.2–59.5	

^1^ Interquartile Range.

**Table 4:** Numerical values of the parameters related to the second heart sound.

Subject ID	R-S_2,A_		R-S_2,P_		S_2,A_-S_2,P_	
	Value (ms)	Std (ms)	Value (ms)	Std (ms)	Value (ms)	Std (ms)
subj_01	360.2	4.3	382.5	12.4	22.4	12.8
subj_02	328.0	38.4	346.3	40.6	18.3	20.4
subj_03	337.8	9.2	348.1	13.9	10.3	11.3
subj_04	429.7	6.7	450.1	18.2	20.4	14.9
subj_05	353.3	33.4	368.3	37.5	15.0	16.7
subj_06	398.8	5.4	447.8	12.6	49.0	11.6
subj_07	372.0	4.3	407.8	14.7	35.8	14.2
subj_08	392.3	7.1	413.2	17.2	20.8	13.3
subj_09	342.8	8.8	386.4	17.9	43.6	13.4
subj_10	371.1	60.4	390.4	60.4	19.3	16.0
subj_11	379.7	9.6	391.6	12.1	11.9	5.8
subj_12	391.6	7.5	451.9	31.1	60.3	26.6
subj_13	361.7	16.6	393.6	24.5	31.9	18.2
subj_14	394.9	11.2	423.0	18.7	28.1	16.9
subj_15	351.1	10.0	392.8	13.0	41.6	12.4
subj_16	407.3	27.2	433.4	37.1	26.1	22.4
subj_17	360.8	19.6	384.7	26.6	23.9	19.7
subj_18	345.8	10.2	370.1	16.7	24.3	13.1
subj_19	340.7	39.7	360.3	48.4	19.6	25.0
subj_20	407.6	8.1	437.8	13.6	30.2	13.0
subj_21	395.1	21.0	424.2	31.9	29.1	21.8
subj_22	382.1	9.7	403.3	12.2	21.2	15.0
subj_23	352.1	9.0	387.6	36.6	35.6	38.6
subj_24	355.0	8.4	389.4	16.4	34.4	15.2
**Mean**	371.3		399.4		28.0	
**Median**	366.4		392.2		25.2	
**Std**	21.2		27.6		18.2	
**IQR** ^1^	42.9		40.8		15.4	
**Range 95%**	333.6–417.0		347.4–450.9		11.2–53.8	

^1^ Interquartile Range.

## Supplementary Materials

The following are available online at https://www.mdpi.com/1424-8220/19/8/1868/s1, Signal S1: subj_01.bin, Signal S2: subj_18.bin, Signal S3: subj_23.bin.
